# O-specific polysaccharide confers lysozyme resistance to extraintestinal pathogenic *Escherichia coli*

**DOI:** 10.1080/21505594.2018.1433979

**Published:** 2018-03-19

**Authors:** Yinli Bao, Haobo Zhang, Xinxin Huang, Jiale Ma, Catherine M. Logue, Lisa K. Nolan, Ganwu Li

**Affiliations:** a Department of Veterinary Diagnostic and Production Animal Medicine, College of Veterinary Medicine, Iowa State University, Ames, IA, USA; b Department of Veterinary Preventive Medicine, College of Veterinary Medicine, Nanjing Agricultural University, Nanjing, China; c Shanghai Entry-Exit Inspection and Quarantine Bureau, Shanghai, China; d Department of Veterinary Microbiology and Preventive Medicine, College of Veterinary Medicine, Iowa State University, Ames, IA, USA; e State Key Laboratory of Veterinary Biotechnology, Harbin Veterinary Research Institute, Chinese Academy of Agricultural Sciences, Harbin, China

**Keywords:** Extraintestinal pathogenic *E. coli* (ExPEC), neonatal meningitis-associated *E. coli* (NMEC), lysozyme resistance, lipopolysaccharide (LPS), O-specific polysaccharide

## Abstract

Extraintestinal pathogenic *Escherichia coli* (ExPEC) is the leading cause of bloodstream and other extraintestinal infections in human and animals. The greatest challenge encountered by ExPEC during an infection is posed by the host defense mechanisms, including lysozyme. ExPEC have developed diverse strategies to overcome this challenge. The aim of this study was to characterize the molecular mechanism of ExPEC resistance to lysozyme. For this, 15,000 transposon mutants of a lysozyme-resistant ExPEC strain NMEC38 were screened; 20 genes were identified as involved in ExPEC resistance to lysozyme—of which five were located in the gene cluster between *galF* and *gnd*, and were further confirmed to be involved in O-specific polysaccharide biosynthesis. The O-specific polysaccharide was able to inhibit the hydrolytic activity of lysozyme; it was also required by the complete lipopolysaccharide (LPS)-mediated protection of ExPEC against the bactericidal activity of lysozyme. The O-specific polysaccharide was further shown to be able to directly interact with lysozyme. Furthermore, LPS from ExPEC strains of different O serotypes was also able to inhibit the hydrolytic activity of lysozyme. Because of their cell surface localization and wide distribution in Gram-negative bacteria, O-specific polysaccharides appear to play a long-overlooked role in protecting bacteria against exogenous lysozyme.

## Introduction

Lysozyme is a key player in the innate immune system, secreted by various tissues [1] and cells—including neutrophils and macrophages [2]—and found at high concentrations in the mucosal surface fluids such as the serum, saliva, sweat, and tears. Lysozyme functions as an *N*-acetylmuramide glycanhydrolase to cleave 1,4-beta-linkages between *N*-acetylmuramic acid (NAM) and *N*-acetylglucosamine (NAG) in the bacterial peptidoglycan, which leads to the lysis of the bacterial cell wall [3]. Lysozyme may also exert a bactericidal effect, via a non-enzymatic mechanism, with its cationic antimicrobial peptide activity and hydrophobic properties inducing cell death via membrane perturbation [4-8]. In addition, the muramidase activity of lysozyme may be involved in the modulation of the immune response and inflammation.

Every attack triggers a counter-attack in the world of microorganisms, hence, it is not surprising that bacteria have developed sophisticated strategies to resist the activity of lysozyme. Gram-positive bacteria, such as *Streptococcus pneumoniae* and *Staphylococcus aureus*, can increase the resistance to lysozyme by modifying their peptidoglycan [9,10]. Well-known modifications include *N*-deacetylation of NAG, O-acetylation of NAM, *N*-glycolylation of NAM, and, more recently, O-acetylation of NAG [11,12]. Gram-negative bacteria are surrounded by a double cell wall that renders them naturally impermeable to lysozyme, and therefore, are usually thought to be insensitive to its effects; however, chemical modifications that render the peptidoglycan lysozyme-resistant have also been reported. Additionally, Gram-negative bacteria produce highly specific and potent proteinaceous lysozyme inhibitors [10]. The first known lysozyme inhibitor, Ivy (inhibitor of a vertebrate lysozyme), was identified in *Escherichia coli* strain MG1655 in 2001 [13]. Later, additional periplasmic and/or membrane-bound lysozyme inhibitors were identified and characterized, including PliC, MliC [14], PliG [15], PliI [16], and Tsi3 [17].

In the course of exploratory investigations in our laboratory, we have discovered that ExPEC strains are more resistant to lysozyme than the *E. coli* strain MG1655. The currently known mechanisms of lysozyme resistance cannot explain this difference, since the proteinaceous lysozyme inhibitors characterized to date are encoded by both pathogenic and commensal *E. coli*. In addition to those identified proteinaceous inhibitors, it has been also reported that *E. coli* lipopolysaccharide (LPS) can bind to lysozyme and inhibit its enzymatic activity [18]; however, the underlying mechanisms are still elusive. We addressed the molecular basis of our initial observations in the current study. We determined that LPS from *E. coli* MG1655 is truncated and lacks the O-specific polysaccharide. The O-specific polysaccharide of ExPEC inhibited the hydrolytic activity of lysozyme. Further, the O-specific polysaccharide was also required by the complete lipopolysaccharide (LPS)-mediated protection of ExPEC against the bactericidal activity of lysozyme.

## Results

### ExPEC strains are more resistant to lysozyme than nonpathogenic E. coli K-12

**Figure 1.**
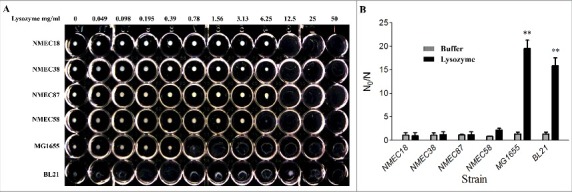
Lysozyme sensitivity of clinical ExPEC isolates and laboratory *E. coli* strains. (A) The densities of six *E. coli* strains in the late log phase of growth (OD_600_ = 2.0) were adjusted to 10^8^ CFU/mL, and the cells incubated with different lysozyme concentrations (0–50 mg/mL) in a 96-well microtiter plate. The lysozyme sensitivity was determined based on MLC, which was the lowest concentration of lysozyme to lyse *E. coli* cells following a 24-h incubation at 37 °C. (B) The *in vitro* lysozyme killing assay. The degree of bacterial lysozyme sensitivity was calculated by dividing the CFU number prior to treatment by the CFU number after a 24-h exposure to lysozyme (N_0_/N). Data represent the mean ± standard deviation (SD) from three independent experiments. **P < 0.01 by one-way ANOVA.

We first established a rapid method to determine the resistance of *E. coli* to lysozyme to facilitate strain screening. *E. coli* cells (1 × 10^8^) were washed three times with phosphate-buffered saline (PBS) , re-suspended in 1 mL of PBS, incubated with different concentrations of lysozyme (0–50 mg/mL) at 37 °C, and then the minimal lytic concentration (MLC) was determined 24 h later (Fig. 1A). Notably, the four tested ExPEC strains were more resistant to lysozyme than nonpathogenic *E. coli* K-12 MG1655 and the laboratory strain BL21. The lysozyme MLC values of the ExPEC strains (NMEC18, NMEC38, NMEC87, and NMEC58) ranged from 6.25 to 12.5 mg/mL, while the lysozyme MLC values of BL21 and MG1655 strains ranged from 0.049 to 0.78 mg/mL (Fig. 1A). This suggested that ExPEC strains were 8- to 16-fold more resistant to lysozyme than *E. coli* K-12 MG1655. These *E. coli* strains were further evaluated by an *in vitro* lysozyme killing assay, where their sensitivity levels to lysozyme were calculated by N_0_/N, where N_0_ and N were the colony counts before and 24-h after incubation. The lysozyme susceptibility test confirmed that all four tested ExPEC strains were significantly (*P* < 0.01) more resistant to lysozyme than MG1655 and BL21 strains (Fig. 1B).

### Identification of NMEC38 strain genes involved in the resistance to lysozyme

A transposon mutant library was constructed using the mini-Tn*5* transposon system in strain NMEC38, one of the lysozyme-resistant ExPEC strains; the library contained 15,000 individual transposon mutants. The mutant library was screened as described in the Materials and methods section, to identify mutants with decreased resistance to lysozyme. In total, 25 mutant strains that showed a reproducible and substantial decrease in lysozyme resistance were identified in the screening assay. The insertion sites of mini-Tn*5* in the selected 25 mutants were determined by amplifying their flanking DNA regions in arbitrarily primed polymerase chain reactions (PCR), followed by sequencing of the amplified DNA products (between 150- and 750-bp long). For sequence analyses, BLASTX or BLASTN (https://blast.ncbi.nlm.nih.gov/Blast.cgi) hits with the highest scores and lowest *e* values were identified. Of the 25 mutants, 20 harbored transposon insertions in different genes (Table 3). The identified genes included genes encoding enzymes involved in LPS biosynthesis, central metabolism, and prophage function; genes with putative regulatory functions and Tripartite ATP-independent periplasmic transport (TRAP); and genes with unknown function.

**Table 1.** *E. coli* strains and plasmids used in this study.

Strain or plasmid	Genotype or description	Source
Wild type strains		
NMEC38	Serotype: O18	Laboratory stock [32]
NMEC18	Clinical isolate	Laboratory stock [32]
NMEC58	Clinical isolate	Laboratory stock [32]
NMEC87	Clinical isolate	Laboratory stock
S17-1 λpir	RK2 *tra* regulon, *pir*, host for *pir-*dependent plasmids	Laboratory stock
MG1655	*F-λ- ilvG- rfb-50 rph-1*	Laboratory stock
*E. coli* DH5α	Cloning host for maintaining the recombinant plasmids	Laboratory stock
*E. coli* BL21	*fhuA2 [lon] ompT gal [dcm] ΔhsdS*	Laboratory stock
Mutant strains		
N380	NMEC38 Δ *ECOK1_2260*	This study
N381	NMEC38 Δ *ECOK1_2261*	This study
N383	NMEC38 Δ *ECOK1_2263*	This study
N384	NMEC38 Δ *ECOK1_2264*	This study
N385	NMEC38 Δ *ECOK1_2265*	This study
N386	NMEC38 Δ*rfbD*	This study
N387	NMEC38 Δ *ECOK1_2261–2264*	This study
*ΔECOK1_3365-neuD*	NMEC38 *ΔECOK1_3365-neuD*	This study
Complemented strains		
N380C	N380 with plasmid p380	This study
N381C	N381 with plasmid p381	This study
N383C	N383 with plasmid p383	This study
N384C	N384 with plasmid p384	This study
N385C	N385 with plasmid p385	This study
N386C	N386 with plasmid p386	This study
N387C	N387 with plasmid p387	This study
Plasmids		
pKD3	Template for λ-Red Chl^r^ cassette	[35]
pKD4	Template for λ-Red Kan^r^ cassette	[35]
pCP20	Encodes FLP recombinase for the removal of resistance cassette	[35]
pKD46	λ-Red recombinase expression	^35^
pGEN-MCS	Low-copy plasmid for complementation	[42]
pGEN/pbla	MCS was replaced by the promoter region of ampicillin	[36]
pGEN/pcm	MCS was replaced by the promoter region of chloramphenicol	This study
p380	pGEN/pcm carrying *2260* coding region	This study
p381	pGEN/pbla carrying *2261* coding region	This study
p383	pGEN/pbla carrying *2263* coding region	This study
p384	pGEN/pbla carrying *2264* coding region	This study
p385	pGEN/pcm carrying *2265* coding region	This study
p386	pGEN/pcm carrying *rfbD* coding region	This study
P387	pGEN/pbla carrying coding regions from *2261* to *2264*	This study

**Table 2.** Primers used in this study.

Primer	Sequence (5′−3′)	Target gene, locus, or application
Primers for arbitrary PCR		
P6	CCTAGGCGGCCAGATCTGAT	For transposon identification
P9	CGCAGGGCTTTATTGATTC	For transposon identification
Arbi2	GGCCACGCGTCGACTAGTAC	For transposon identification
Arbi5	GGCCACGCGTCGACTAGTAC(N)_10_TACNG	For transposon identification
Primers for gene deletion^a^^,^^b^		
D380F	TTTCTGACACTCATATTAATTATGAGTGGTACGTTTGGTAAACGGTAAACTATTAT*gtgtaggctggagctgcttcga*	ECOK1_*2260*
D380R	TGTAATTTTATTTTCACTTTGAAAAACCTGTTCTTTTTTAACTTTTCGGTTTCAT*catatgaatatcctccttag*	
D381F	AGATGCTAATTAGATATTTGCAATGTTGTTATTATGAGAAAATAAAATGA*gtgtaggctggagctgcttcga*	ECOK1_*2261*
D381R	CAATTGAGATTGAATTAAATTCAAACAAAAGACACGTTCCAAATATAAAT*catatgaatatcctccttag*	
D383F	GTACCATCTTACTATGGCGATTTACAGTAATATGCAAACCAGTACAGTAA*gtgtaggctggagctgcttcga*	ECOK1_*2263*
D383R	CTTGATAAAGTATGTTGCCGATTAAAAGTAGGTGTAAGTATTGAAATCAT*catatgaatatcctccttag*	
D384F	AAATGCAAGTTAATAACTCATGGCTTTATTTGGGTAGGTGACAATTTATA*gtgtaggctggagctgcttcga*	ECOK1_*2264*
D384R	GCCTCGCCATTGTAGGTGGCCATTAGAATGGTTACTGTACTGGTTTGCAT*catatgaatatcctccttag*	
D385F	TAAATGCGCCAACATTTAAGAAAATATCGAGTAATGAGTATTTTAAATGA*gtgtaggctggagctgcttcga*	ECOK1_*2265*
D385R	TTGTCACCTACCCAAATAAAGCCATGAGTTATTAACTTGCATTTTGAATT*catatgaatatcctccttag*	
D386F	GTGGTGCCTATCAATCGTGGATTGAACAGAACTATGAGGGCCGCCACTAA*gtgtaggctggagctgcttcga*	*rfbD*
D386R	TTCCTTTTAATTCATCTTGTTCCACCATCACGAACAAGATGCAAAAACTA*catatgaatatcctccttag*	
Primers for deletion confirmation		
C380F	GGTTTTCGGAATCGTGAGCG	ECOK1_*2260*
C380R	CTTTCGATGTTGAGCGCGAG	
C381F	AACTCCCGATGCCATAAA	ECOK1_*2261*
C381R	CTCTGTTCCCAGCCCAAT	
C383F	CTGAAACGCTAGTAACGA	ECOK1_2263
C383R	TCGAATCTTCGCCTTGTC	
C384F	GGTATTGCGTGGTCAGTG	ECOK1_*2264*
C384R	ACATAAGGTATTTCGGGAGA	
C385F	CAACAAGCAGAGCGAAGC	ECOK1_*2265*
C385R	TACCCGTATAGCCCTCCA	
C386F	TGGTCCTTATCATTTCCC	*rfbD*
C386R	TTAACTTAGGCAGGTCGTG	
C1	TTATACGCAAGGCGACAAGG	*cm*
C2	GATCTTCCGTCACAGGTAGG	
K1	CAGTCATAGCCGAATAGCCT	*Km*
K2	CGGTGCCCTGAATGAACTGC	
Kt	CGGCCACAGTCGATGAATCC	
Primers for complementation experiments^c^		
p380F	ACGC GTCGAC ATGACAGCTAGAACAACTAA	ECOK1_*2260*
p380R	TGCG GTCGAC TTAATTATCATATAGCTGTCTG	
p381F	ACGCGTCGACATGAAAATACTATTTGTCATT	ECOK1_*2261*
p381R	TGCGGTCGACCTACCTTTCATGTTTTGAGCA	
p383F	ACGCGTCGACATGATTTCAATACTTACACCT	ECOK1_*2263*
p383R	TGCGGTCGACTCATTTTATTTTCTCATAATA	
p384F	ACGCGTCGACATGCAAACCAGTACAGTAACC	ECOK1_*2264*
p384R	TGCGGTCGACTCATTCGCTAAATTTTCTCC	
p385F	ACGCGTCGACATGATATATATATTAACTTT	ECOK1_*2265*
p385R	TGCGGTCGACTTACTGTACTGGTTTGCATA	
p386F	ACGCGTCGACATGAGTTTAATCAAAAACA	*rfbD*
p386R	TGCGGTCGACTTATTTATTAATATACTTACCG	
pPro384F	ACGCGTCGACTCAAGAGCCTTATTTCCAA	ECOK1_*2261* to *2264*

a The underlined sequences are homologous to the pKD3 or pKD4 vector sequence flanking the Cm^r^ or Km^r^ genes

b Italicized sequences are homologous to the target gene flanking sequence.

c The underlined sequences are the enzyme digestion sites.

**Table 3.** Genetic loci disrupted by mini-Tn*5* in the derivatives of strain NMEC38 Nal^r^.

Recombinant class	Recombinant	MLC^a^ (mg/mL)	Ref. strain	Gene/locus tag	Accession No^b^.	Identity (%)	Putative function
LPS synthesis	F72	5	IHE3034	*rfaJ*	ADE89653	97	Lipopolysaccharide 1,2-glucosyltransferase
	M62	6.25	IHE3034	ECOK1_2260	ADE92608	100	Glycosyl transferase, group 1 family protein
	U424	3.125	IHE3034	*rfbD*	ADE89399	97	dTDP-4-dehydrorhamnose reductase
	U475	6.25	IHE3034	ECOK1_2263	ADE92801	91	Glycosyl transferase, group 2
	k262	1.56	IHE3034	ECOK1_2264	ADE89668	97	Putative membrane protein
	F172	0.3125	IHE3034	ECOK1_2261	ADE91992	98	Glycosyl transferase, group 1 family protein
	J399	1.56				98	
	L446	0.78				98	
	T358	6.25	IHE3034	ECOK1_2265	ADE89127	87	Putative O-antigen transporter
	V66	6.25				94	
	U504	6.25	IHE3034	*rfaI*	ADE88840	99	Lipopolysaccharide 1,3-galactosyltransferase
	V501	6.25				86	
	J42	6.25	IHE3034	ECOK1_4065	ADE89362	94	Lipid A-core surface O-antigen polymerase
	L208	3.125				98	
Metabolism	F185	5	IHE3034	*pepA*	ADE89150	98	Leucyl aminopeptidase
	G295	5	IHE3034	ECOK1_2199	ADE89584	99	Glycosyl transferase, group 2 family protein
	I244	6.25	IHE3034	*allD*	ADE89059	95	Ureidoglycolate dehydrogenase
	K240	3.125	IHE3034	*Pgi*	ADE90626	95	Glucose-6-phosphate isomerase
	T352	6.25	IHE3034	*dsbA*	ADE89344	98	Thiol:disulfide interchange protein DsbA
	V153	6.25	IHE3034	*galU*	ADE90279	90	UTP-glucose-1-phosphate uridylyltransferase
Prophage	F199	5	CFT073	*intC*	AE016769	92	Putative prophage integrase
Regulator	B54	6.25	IHE3034	*dhaR*	ADE89325	91	PTS-dependent Dihydroxyacetone kinase operon regulatory protein
Transporter	O159	1.56	IHE3034	ECOK1_4026	ADE92303	92	TRAP transporter, DctM family
Unknown	G413	6.25	pECOS88	ECS88_p0136	CAQ87198	98	Hypothetical protein
	H157	6.25	E.coli W	WFL_20590	AFH13760	96	Putative repressor protein C

a MLC, minimal lytic concentration

### Novel genes involved in O-antigen biosynthesis contribute to ExPEC resistance to lysozyme

**Figure 2.**
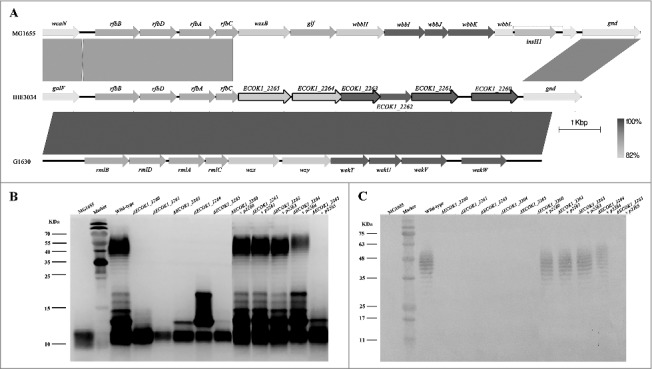
Novel genes involved in the O-antigen biosynthesis contribute to ExPEC resistance to lysozyme. (A) Comparison of the O-antigen cluster in IHE3034 (NC_017628.1), MG1655 (NC_000913), and G1630 (GU299793) strains. Genes designated by purple are involved in sugar biosynthesis; green, genes involved in the O-antigen processing; blue, genes encoding glycosyltransferase enzymes; and black outline, genes that have been found to be associated with lysozyme resistance in the current study. The linear representation of the genetic comparison was generated using Easyfig version 2.1. (B) A silver-stained polyacrylamide gel after SDS-PAGE (top) and generalized LPS structures (bottom). (C) Western blotting profiles of LPS probed with the anti-O18 serum. Compared with the wild-type strain, the banding pattern in the O-antigen region was altered in all mutant strains. The complemented strains had similar banding patterns to the wild type, except for the complemented strain Δ*ECOK1_2265.* Rha: rhamnose; Gal: galactose; Glc: glucose; Kdo: 3-deoxy-d-manno-oct-2-ulosonic acid; Hep: l-glycero-d-manno-heptose; GlcNAc: *N*-acetylglucosamine.

In eight mutant strains, the transposon disrupted either of five genes homologous to *ECOK1_2260*, *ECOK1_2261*, *ECOK1_2263*, *ECOK1_2264*, and *ECOK1_2265* [19]; these mutant strains were among the most sensitive to lysozyme (Table 3). Sequence analysis of these five genes revealed that they are located on the O-antigen island (between the *galF* and *gnd* genes; Fig. 2A) [19], suggesting that they may be novel genes involved in the O-antigen biosynthesis. To determine their functions, five non-polar mutant strains and their respective complemented strains were constructed. LPS of the five mutant strains, five complemented strains, the wild-type strain (NMEC38), and the laboratory strain MG1655 were isolated, and analyzed by sodium dodecyl sulfate-polyacrylamide gel electrophoresis (SDS-PAGE) and silver-staining. Compared with the wild-type strain, all mutant strains showed altered banding patterns in the O-antigen regions (Fig. 2B, 2C), suggesting that the O-antigen chain was truncated, suggesting that all five genes play a role in the O-antigen biosynthesis. These analyses also demonstrated that the O-antigen chain in *E. coli* K-12 strain MG1655 was truncated. When the genes were reintroduced into the respective mutant strains, the LPS of four complemented strains showed a similar banding pattern as the wild-type parent, confirming that genes *ECOK1_2260*, *ECOK1_2261*, *ECOK1_2263*, and *ECOK1_2264* play a role in the O-antigen biosynthesis.

**Figure 3.**
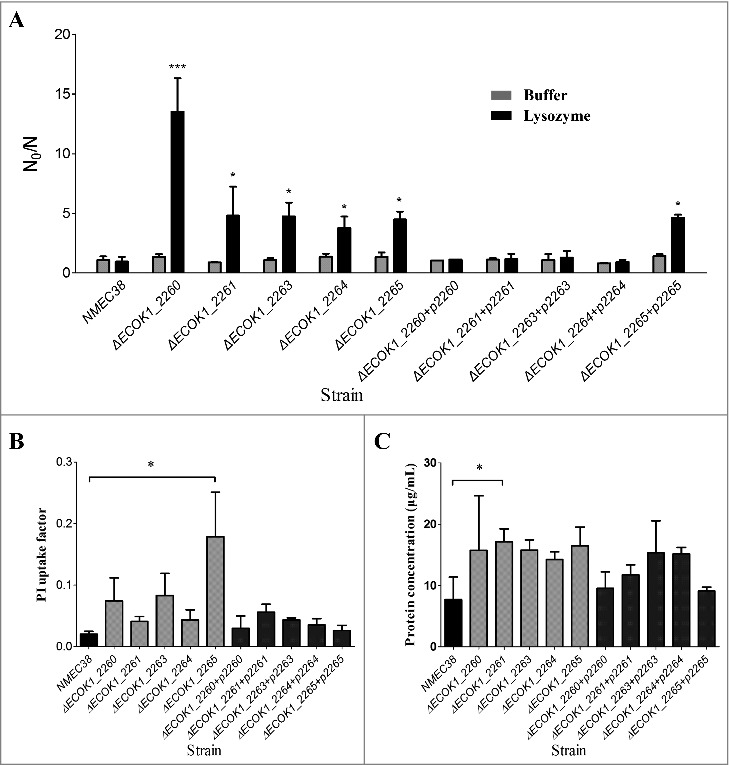
The *in vitro* lysozyme killing assay and membrane integrity tests. (A) Lysozyme sensitivity of the wild-type, mutant, and complemented strains was determined by an *in vitro* killing assay. The strain compromised viability levels (N_0_/N) under the indicated conditions were analyzed as in Fig. 1B. (B) Membrane integrity of the wild-type, mutant, and complemented strains determined by PI staining. (C) Membrane integrity determined by a protein leakage assay. Data represent the mean ± standard deviation (SD) from three independent experiments. *P < 0.05, **P < 0.01 by one-way ANOVA.

Wild-type, mutant, and complemented strains were then evaluated by an *in vitro* lysozyme killing assay to confirm the role of these genes in lysozyme resistance (Fig. 3A). In the absence of lysozyme, all mutant strains survived very well in 1 mM Tris-HCl (pH 7.2), with N_0_/N values ranging from 0.82 to 1.37, which were not significantly different from those for the wild-type and complemented strains. After a 24-h incubation with lysozyme (5 mg/mL), all mutants showed a substantial reduction in viability, with an almost 10-fold reduction compared to the wild type, while the complemented strains recovered to the wild-type level. These results confirmed that these novel genes involved in O-antigen biosynthesis contributed to the ExPEC resistance to lysozyme.

### Membrane integrity is weakened in ExPEC mutants with defects in O-antigen biosynthesis

Propidium iodide (PI) staining was employed to examine whether the deletion of newly identified genes involved in the O-antigen biosynthesis would affect *E. coli* membrane permeability [20,21]. Bacterial cells with intact membranes are impermeable to charged fluorescent dyes, such as PI; however, if the membrane integrity is compromised, PI can enter the cell and, by binding to the nucleic acid, render the cell fluorescent. The PI uptake in all mutant strains was increased in comparison with that in the wild type, but the difference was only significant in the mutant strain Δ*ECOK1_2265* (Fig. 3B, *P* < 0.05). A protein leakage assay was performed to further evaluate membrane integrity of the wild-type and O-antigen gene mutant strains [20,21]. Similar to the results of the PI staining assay, all mutant strains showed increased protein leakage; however, the difference with the wild type was significant only in the mutant strain Δ*ECOK1_2261* (Fig. 3C, *P* < 0.05). Taken together, these results suggested that the deletion of the O-antigen synthesis genes affected *E. coli* membrane integrity, but might not have been the major mechanism of the reduced lysozyme resistance of mutants.

### O-specific polysaccharide is necessary for the LPS-mediated protection of ExPEC from the bactericidal activity of lysozyme

**Figure 4.**
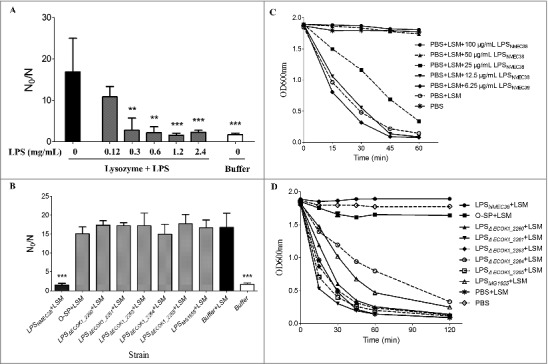
The *in vitro* inhibition of lysozyme activity by LPS and the O-specific polysaccharide from NMEC38 wild type. (A) *In vitro* inhibition of the lysozyme killing activity. To determine the effect of LPS_NMEC38_ on lysozyme killing activity, Δ*ECOK1_2265* strain was incubated with lysozyme in the presence or absence of diluted LPS_NMEC38_ for 24 h. The control cells were incubated in 1 mM Tris-HCl (pH 7.2). Inactivation levels (N_0_/N) were determined after the incubation period. (B) LPS without the O-specific polysaccharide and O-specific polysaccharide alone cannot inhibit the bactericidal activity of lysozyme. Compromised viability (N_0_/N) were determined in the absence and presence of 2.0 mg/mL of LPS or O-SP. (C) *In vitro* inhibition of lysozyme hydrolysis activity by different concentrations of LPS. *M. lysodeikticus* cell lysis was determined as the decrease in optical density at 600 nm (OD_600_) over time, in the presence of 4 μg/mL of lysozyme, and in the absence or presence of LPS from the wild type, NMEC38. (D) The ability of LPS lacking the O-specific polysaccharide to inhibit the hydrolytic activity of lysozyme is substantially reduced. *M. lysodeikticus* cells were suspended in the presence of 4 μg/mL of lysozyme in the absence or presence of 100 μg/mL of LPS from wild-type, mutant, and MG1655 strains, and the experiment was performed as in (C). Data represent the mean ± standard deviation (SD) from three independent experiments. *P < 0.05; **P < 0.01; ***P < 0.001 by one-way ANOVA. LSM, Lysozyme; O-SP, O-specific polysaccharide.

To investigate the mechanism(s) whereby the O-antigen synthesis genes contribute to lysozyme resistance of ExPEC, we tested whether ExPEC LPS could inhibit the bactericidal activity of lysozyme. Complete LPS was extracted from NMEC38 (wild type) by a hot phenol-water method [18,22]. The mutant strain Δ*ECOK1_2265*, which was the most lysozyme-susceptible mutant strain, was cultured to the late exponential phase (10^9^ CFU/mL) and used in *in vitro* lysozyme killing assay. The mutant strain Δ*ECOK1_2265* was incubated with 5 mg/mL of lysozyme and different concentrations of wild-type LPS in 1 mM Tris-HCl (pH 7.2); the survivors were enumerated after 24 h. In the absence of wild-type ExPEC LPS, the lysozyme resulted in an approximately 16-fold bacterial reduction and killed most mutant cells; in contrast, upon the addition of 0.12 mg/mL of LPS, the lysozyme resulted in only a 10.8-fold bacterial reduction (Fig. 4A). When more than 0.3 mg/mL of LPS was added, most mutant cells survived the lysozyme treatment, showing a 1.57- to 2.3-fold bacterial reduction (Fig. 4A), suggesting that wild-type LPS could indeed inhibit the bactericidal activity of lysozyme.

Since LPS consists of a lipid A unit, core oligosaccharide and an O-specific polysaccharide chain, the ability of LPS lacking the O-specific polysaccharide and the O-specific polysaccharide alone to inhibit the bactericidal activity of lysozyme was next tested. LPS lacking the O-specific polysaccharide was extracted from mutant strains Δ*ECK1_2260*, Δ*ECK1_2261*, Δ*ECK1_2263*, Δ*ECK1_2264*, and Δ*ECK1_2265*; and the O-specific polysaccharide was purified from NMEC38 wild type. The ability of LPS lacking the O-specific polysaccharide and the O-specific polysaccharide alone to inhibit the bactericidal activity of lysozyme was significantly (P < 0.001) reduced compared to complete LPS (Fig. 4B). These results indicated that the O-specific polysaccharide alone cannot inhibit the bactericidal activity of lysozyme but is necessary for the LPS-mediated protection of ExPEC against this activity.

### O-specific polysaccharide from NMEC inhibits the hydrolytic activity of lysozyme

LPS from NMEC38 wild type was further examined by a turbidity assay described by Callewaert [14] to determine whether it could inhibit the hydrolytic activity of lysozyme. When *Micrococcus lysodeikticus* ATCC 4698 was incubated with 4 μg/mL of lysozyme in the absence of LPS, the OD600 value of the reaction mixture dropped close to zero after 2 h, suggesting that *M. lysodeikticus* ATCC 4698 cells were almost entirely lysed (Fig. 4C). When LPS (>50 μg/mL) was included in the reaction mixture, the OD600 value of the reaction remained constant, with a turbidity similar to that of the negative control incubated in the absence of lysozyme (Fig. 4C), suggesting that the lytic activity of lysozyme was completely inhibited. These results indicated that LPS from NMEC38 could inhibit the hydrolytic activity of lysozyme.

We next investigated whether LPS lacking the O-specific polysaccharide or the O-specific polysaccharide alone could inhibit the hydrolytic activity of lysozyme. The experiment (Fig. 4D) revealed that the ability of LPS that lacked the O-specific polysaccharide (purified from ExPEC mutant strains) to inhibit the hydrolytic activity of lysozyme was significantly reduced compared to the complete LPS. Similarly, LPS purified from *E. coli* K-12 strain MG1655 was unable to inhibit that activity. The O-specific polysaccharide from NMEC38 wild-type almost entirely inhibited the hydrolytic activity of lysozyme, emphasizing the pronounced differences between LPS lacking the O-specific polysaccharide and LPS from the *E. coli* K-12 strain MG1655.

### LPS and the O-specific polysaccharide from ExPEC can directly interact with lysozyme

**Figure 5.**
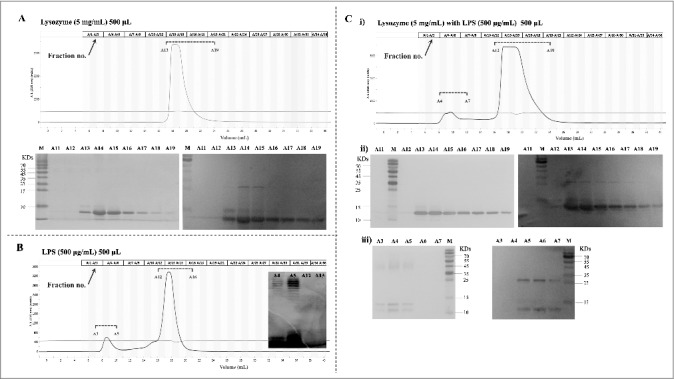
Analysis of LPS_NMEC38_ and lysozyme by gel filtration chromatography. (A) In the absence of LPS, lysozyme was eluted in fractions no. 13–19. SDS-PAGE and western blotting were used to detect the eluted lysozyme. (B) In the absence of lysozyme, LPS was eluted in two peaks, as determined by photometry, but only the first peak contained LPS, as indicated by SDS-PAGE (LPS silver-staining) (fractions 3–5). (C) Co-incubation of lysozyme with LPS resulted in the presence of lysozyme in the forward-shifted peak that contained fractions A4–A7. i) Gel filtration chromatography analysis of the lysozyme-LPS complex. ii) Presence of lysozyme in the peak containing fractions A13–A19, as demonstrated by SDS-PAGE (LPS silver-staining) and western blotting (with anti-lysozyme antibody). iii) Presence of lysozyme in the pronouncedly forward-shifted peak containing fractions A4–A7 (lysozyme presence in the peaks containing fractions 13–19 in Fig. 5A) shown by SDS-PAGE (LPS silver-staining) and western blotting (with anti-lysozyme antibody).

The direct interaction of LPS with lysozyme was examined by gel filtration chromatography. In this assay, LPS and/or lysozyme were pre-incubated for 30 min in a binding buffer and resolved on an Enrich SEC 650 (10/300) gel filtration column, as specified in the Materials and methods section. The elution profile was then analyzed by measuring absorbance at 215 nm and western blotting (with a specific anti-lysozyme antibody). In the absence of LPS, lysozyme was eluted in fractions 13–19 (Fig. 5A). In the absence of lysozyme, LPS eluted as two peaks; however, only the first peak contained LPS, as revealed by western blotting (fractions 3–5, Fig. 5B). Similar observations have been reported by Miki et al [22]. Nevertheless, co-incubation of lysozyme with LPS resulted in the occurrence of lysozyme in both peaks in fractions A4–A7 and 13–19 (Fig. 5C). The lysozyme-LPS complex in the pronouncedly forward-shifted peak A4–A7 was confirmed by western blotting (with anti-lysozyme antibody) and LPS (by silver-staining) (Fig. 5C). The results strongly suggested that LPS bound lysozyme directly.

**Figure 6.**
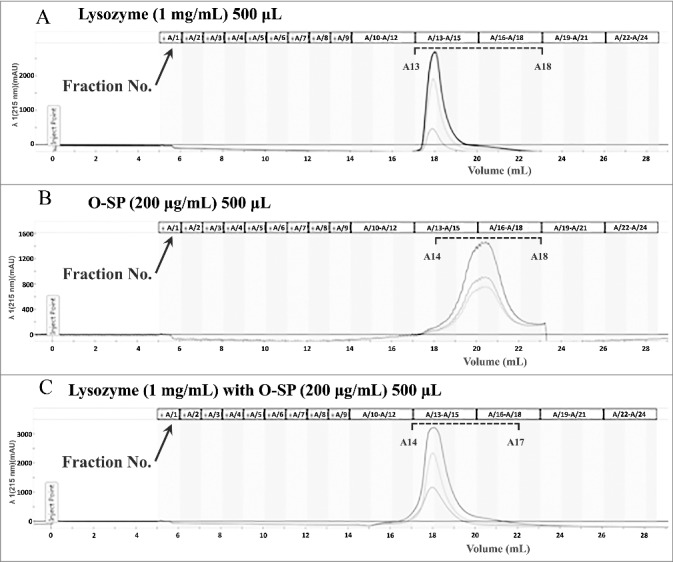
The O-specific polysaccharide of ExPEC can directly bind to lysozyme. (A) Gel filtration chromatogram of lysozyme. (B) Gel filtration chromatogram of the O-specific polysaccharide. (C) Co-incubation of lysozyme with the O-specific polysaccharide resulted in only one peak in the chromatogram; the peak was pronouncedly forward-shifted in comparison with the peak of the O-polysaccharide in (B). O-SP, O-specific polysaccharide.

The interaction of the O-specific polysaccharide to lysozyme was also analyzed by gel filtration chromatography. The O-specific polysaccharide isolated from NMEC38 was pre-incubated in the presence or absence of lysozyme, and then loaded onto the Enrich SEC 650 (10/300) column. The elution profile was analyzed by monitoring *A*215. In the lysozyme only-sample, lysozyme was eluted in fractions number 13–18 (Fig. 6A). As shown in Fig. 6B, in the absence of lysozyme, the O-polysaccharide was eluted in fractions 14–18. Co-incubation of lysozyme with the O-polysaccharide resulted in only one peak, which was pronouncedly forward-shifted compared to the peak of the purified O-polysaccharide, confirming the interaction of O-polysaccharide to the lysozyme (Fig. 6C).

### LPS from ExPEC strains with different O serotypes inhibit the hydrolytic and bactericidal activities of lysozyme

**Figure 7.**
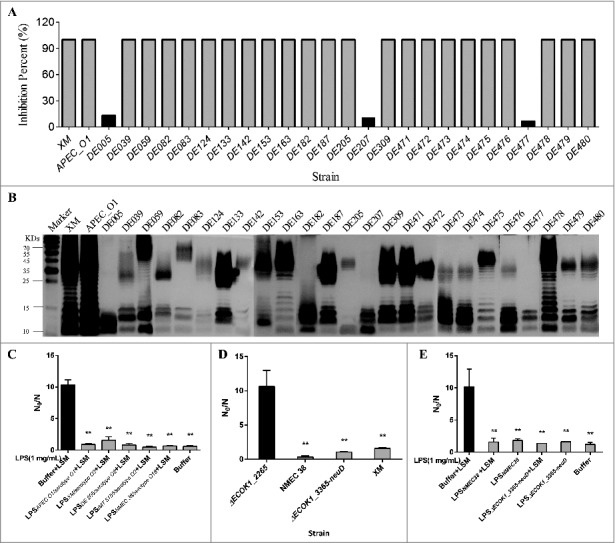
(A) Percent inhibition of the hydrolytic activity of lysozyme. Strains that harbor intact LPS (24) completely inhibited the hydrolytic activity of lysozyme, while the inhibition was almost unnoticeable with strains lacking the O-specific polysaccharide; (B) LPS profiles of 27 ExPEC strains analyzed by SDS-PAGE and silver-staining; (C) the purified LPS from ExPEC with O1, O2, and O18 could protect mutant strain ECOK1_2265 from the bactericidal activity of lysozyme; (D) deletion of capsule biosynthesis genes (ΔECOK1_3365-neuD) from wild type strain did not significantly reduce the ExPEC's resistance to lysozyme, while deletion of O antigen biosynthesis gene (ΔECOK1_2265) significantly reduced ExPEC's resistance to lysozyme, XM: Extraintestinal pathogenic *E. coli* wild type strain ExPEC XM with serotype of O2 (control); and (E) purified LPS from mutant strain with capsule biosynthesis gene (ΔECOK1_3365-neuD) deletion showed that capsule polysaccharides were not necessary for the LPS-mediated protection of ExPEC from the bactericidal activity of lysozyme. Data represent the mean ± standard deviation (SD) from three independent experiments. *P < 0.05; **P < 0.01; by one-way ANOVA. LSM, Lysozyme.

To examine if LPS from strains with different O serotypes could inhibit the hydrolytic activity of lysozyme, additional 27 ExPEC strains with different O serotypes (including O1, O2, O18, O8, and untypeable serotypes) isolated at different times and from different regions [23], were examined. LPS from 24 strains (89.7%) was able to completely inhibit the hydrolytic activity of lysozyme (Fig. 7A); however, this effect was almost abolished in the case of LPS from three ExPEC strains (10.3%) with untypeable O serotypes (DE005, DE207, and DE477; Fig. 7A). SDS-PAGE and silver-staining revealed that similar to that in *E. coli* K-12 strain MG1655, LPS from those three strains was truncated and lacked the O-polysaccharide (Fig. 7B). Furthermore, the purified LPS from ExPEC with O1, O2, and O18 could protect mutant strain Δ*ECOK1_2265* from the bactericidal activity of lysozyme (Fig. 7C). To rule out influence of other surface antigens for example capsular antigen, we deleted the capsule genes (*ECOK1_3365-neuD*) and found no significant difference between wild type and the capsule mutant in lysozyme in vitro killing assay. As the control, deletion of O antigen biosynthesis gene (*ΔECOK1_2265*) significantly reduced ExPEC's resistance to lysozyme (Fig. 7D). We further purified LPS from capsule deleted mutant strain (Δ*ECOK1_3365-neuD*) and showed that capsule polysaccharides were not necessary for the LPS-mediated protection of ExPEC from the bactericidal activity of lysozyme (Fig. 7E).

## Discussion

LPS consisting of a lipid A unit, core oligosaccharide and an O-specific polysaccharide chain is known to interact with lysozyme and inhibit its enzymatic activity [18]. Although the binding of lipid A and a synthetic monosaccharide lipid A analogue to lysozyme has been experimentally confirmed [24], it remains unknown whether lipid A and/or the O-specific polysaccharide indeed inhibit lysozyme activity. In the current study, we identified several genes involved in the synthesis of the O-specific polysaccharide, which contributed to bacterial lysozyme resistance. Deletion of these genes resulted in truncated LPS that lacked the O-polysaccharide, which substantially decreased the resistance of ExPEC mutants to the bactericidal activity of lysozyme. Correspondingly, LPS that lacked the O-specific polysaccharide purified from the constructed mutant strains was characterized by a significantly lower ability to inhibit the bactericidal and hydrolytic activities of lysozyme than the complete LPS purified from the ExPEC wild type. LPS was likely to self-aggregate into supramolecular structures and directly interact with lysozyme, thus inhibit lysozyme's activity [25]. Furthermore, our results also indicated that the purified O-specific polysaccharide alone could inhibit the hydrolytic activity of lysozyme by a direct interaction with the enzyme. To the best of our knowledge, this is the first-ever report that O-specific polysaccharide from ExPEC contributes to bacterial lysozyme resistance.

Lysozyme kills Gram-positive bacteria by cleaving the cell wall peptidoglycan. In addition, accumulating evidence suggests that lysozyme can kill both Gram-positive and -negative bacteria also independently of its enzymatic muramidase activity [4,5,7], with its cationic antimicrobial peptide activity and hydrophobic properties playing an important role [8]. We demonstrated that the complete LPS from ExPEC wild type was able to inhibit the bactericidal activity of lysozyme, while neither LPS lacking the O-specific polysaccharide nor the O-specific polysaccharide alone showed this activity. These observations suggest that the O-specific polysaccharide is required for LPS-mediated inhibition of the bactericidal activity of lysozyme, but by itself, the O-specific polysaccharide lacks this activity. On the other hand, the O-specific polysaccharide alone was able to entirely inhibit the enzymatic and lytic activities of lysozyme, independently of the remaining portion of LPS. The different mechanisms whereby LPS inhibits the various activities of lysozyme require further investigation.

Gram-negative bacteria are generally thought to be more resistant to lysozyme than their Gram-positive counterparts. This difference in resistance is primarily ascribed to the different cell envelope architecture of these two groups of bacteria. Gram-positive bacteria have a thick cell wall composed of up to 40 layers of peptidoglycan, which is very sensitive to lysozyme [26], while Gram-negative bacteria typically have only a single layer of peptidoglycan surrounded by an asymmetric membrane bilayer, which is thought to render Gram-negative bacteria naturally impermeable to lysozyme, and thus resistant to its effects [26]. However, recent evidence indicates that lysozyme can interact with the negatively charged membrane lipid bilayers leading to protein aggregation and membrane fusion [27], and can permeabilize the outer and inner membranes of an *E. coli* mutant ML-35p by inducing the formation of large pores [6,28]. This suggests that the physical barrier afforded by the outer membrane of Gram-negative bacteria might not be the main reason responsible for their greater resistance to lysozyme. LPS, with the O-specific polysaccharide, is a unique and common feature and all Gram-negative bacteria, and our findings suggest that this structure might be responsible for the elevated lysozyme resistance of Gram-negative bacteria. The observation that the O-specific polysaccharide-related lysozyme inhibition activity is independent of the *E. coli* serotype (Fig. 7) and that *Salmonella* LPS that contains the O-specific polysaccharide can also inhibit lysozyme hydrolytic activity (Yinli Bao, data not published) further support this hypothesis.

In addition to the novel O-specific polysaccharide biosynthesis genes and other LPS biosynthesis genes, we also identified metabolism, regulatory, and transport genes involved in bacterial resistance to lysozyme. However, the genes for proteinaceous lysozyme inhibitors, such as Ivy [13], MliC (membrane bound lysozyme inhibitor of c-type lysozyme) [14], and PliG (periplasmic lysozyme inhibitor of g-type lysozyme) [15,29], were not identified in our screen. The performed deletion and lysozyme inhibition assays indicated that these “other” genes did not appear to play a significant role in the ExPEC resistance to lysozyme in our in *vitro* model (Yinli Bao, data not shown). Recently, Ivy and its homolog were shown to be potent inhibitors of lytic transglycosylases involved in the biosynthesis and maintenance of peptidoglycan [30]. In addition, *mliC* or its homolog is adjacent to the *anmK* gene in both *E. coli* and *Salmonella*, which encodes an anhydro-NAM kinase involved in the recycling of murein [14,31]. These findings suggest that the true physiological functions of Ivy, MliC, and PliG might be to control excessive activity of endogenous bacterial autolysins, with the inhibition of exogenous lysozyme as a simply fortuitous coincidence. The localization of the proteinaceous inhibitors to the periplasm (MliC is bound to the luminal side of the outer membrane) [13-15,29] rather than to the external milieu further suggests that the O-specific polysaccharide at the bacterial surface may play a much more important role in protecting bacteria against exogenous lysozyme than proteinaceous inhibitors.

In summary, the present study demonstrated that the O-specific polysaccharide from ExPEC contributes to the LPS-mediated inhibition of the bactericidal activity of lysozyme, and the O-specific polysaccharide alone is able to inhibit the hydrolytic activity of lysozyme through a direct interaction with the enzyme. While these findings were only acquired in a single strain, the observed localization to external milieu and wide distribution of O-specific polysaccharide in Gram-negative bacteria suggests that this surface structure might play an under-appreciated role in protecting bacteria against exogenous lysozyme.

## Materials and methods

### Strains and plasmids

Bacterial strains and plasmids used in this study are listed in Table 1. The transposon mutagenesis library and mutants were created in the ExPEC NMEC38 background [32]. Additional 27 ExPEC strains with different O serotypes [23] were used to evaluate the correlation between the structure of LPS and bacterial resistance to lysozyme. All *E. coli* strains were grown at 37 °C in Luria-Bertani (LB) broth and agar. Antibiotics, including nalidixic acid (30 μg/mL), kanamycin (50 μg/mL), ampicillin (50 μg/mL) and chloramphenicol (30 μg/mL), were added when necessary.

### Mutant library construction, screening, and transposon insertion site identification

More details about crucial steps in the genetic studies were provided in supplementary materials. Briefly, a transposon library of 15,000 mutants was generated using the transposon delivery vector pUTmini-Tn*5*(Km), as described previously [33]. All mutants were inoculated in 96-well, U-bottom plates containing 1 mL of sterile LB medium and grown at 37 °C to late exponential phase (OD_600_ = 2.0). After centrifugation at 4,000 × *g* for 10 min, the bacterial pellets were washed twice with PBS and re-suspended in 1 mL of PBS. Next, 50 μL of the suspensions were transferred to new 96-well plates, and 50 μL of lysozyme solution (12.5 mg/mL, Sigma-Aldrich) was added. The mixtures were incubated at 37 °C for 12 h. The lysed mutant strains were selected for gene identification. The transposon-disrupted genes were identified by amplifying their flanking DNA regions in arbitrarily-primed PCR, followed by sequencing of the amplified DNA products (150–750-bp long) [33]. For sequence analyses, BLASTX or BLASTN (https://blast.ncbi.nlm.nih.gov/Blast.cgi) hits with the highest scores and lowest *e* values were reported as previously described [32].

### Recombinant DNA techniques, SDS-PAGE, and western blotting

PCR, DNA ligation, electroporation, and gel electrophoresis were performed according to Sambrook and Russel [34], unless indicated otherwise. All oligonucleotide primers were purchased from Integrated DNA Technologies and are listed in Table 2. All restriction and DNA-modifying enzymes were purchased from New England Biolabs and were used as per the supplier's recommendations. Recombinant plasmids, PCR products, and restriction fragments were purified using QIAquick PCR purification kit or MinElute gel extraction kit (Qiagen), as recommended by the supplier. DNA sequencing was performed at the DNA Facility, Iowa State University (Ames, IA).

Deletion mutants were constructed using the λ red mutagenesis method [35]; all primers used for mutant construction are listed in Table 2. To construct the plasmids for mutant complementation, the target genes and their native promoters were amplified (the primers are listed in Table 2) from ExPEC wild-type strain (NMEC38) and digested by SalI; they were then ligated into SalI-digested plasmid pGEN/pbla [36]. The resulting plasmids were used to complement the corresponding mutants.

SDS-PAGE and silver-staining of LPS were performed according to the protocol described by Tsai and Frach [37]. Western blotting was performed according to Sambrook and Russel [34], using a semidry blotting apparatus, on polyvinylidene difluoride membranes (Thermo Fisher Scientific). The rabbit anti-*E.* *coli* O18 antibody (1:1000), horseradish peroxidase-conjugated goat anti-rabbit secondary antibody (1:5,000, #31466) and the membrane developing DAB kit were purchased from Thermo Fisher Scientific.

### Lysozyme in vitro killing assay

The *in vitro* killing assay was performed as described previously [38]. The killing activity was calculated by N_0_/N, where N_0_ and N were the colony counts before and 24-h after incubation, respectively. To block lysozyme bactericidal activity, 100 μL of serial dilutions of LPS, LPS lacking the O-specific polysaccharide, or the purified O-specific polysaccharide were used. The samples without LPS or lysozyme were used as controls.

### Membrane integrity determinations

Fluorescence PI staining measurement and protein leakage assays were performed to determine the integrity of the bacterial membrane. PI staining assay was performed as described by Garcia-Gonzalez et al. [20,21], and the protein leakage assay was performed as described previously [20,21]. Protein concentrations in reaction supernatants were determined by the BCA protein assay Kit (Pierce).

### LPS purification and preparation of the O-specific polysaccharide

LPS was extracted by the hot phenol-water method as described previously [18,22], with minor modifications. Briefly, 100 mL of bacterial culture was grown to late logarithmic phase (OD_600_ = 2), centrifuged, washed twice with PBS (pH 7.2), and re-suspended in 20 mL of ddH_2_O. An equal volume of hot 90% (v/v) phenol (68 °C) was slowly added to the mixtures, followed by a vigorous shaking at 68 °C for 30 min. Suspensions were then cooled on ice and centrifuged at 2,851 × g for 45 min at 10 °C. Supernatants were transferred to 50-mL conical tubes; ten volumes of cold absolute ethanol and sodium acetate (to final concentration of 0.3 M) were added, and mixed thoroughly. The reactions were stored at -20 °C overnight and then centrifuged at 2,000 × *g* for 10 min at 4 °C. The LPS pellets were finally re-suspended in 1 mL of ddH_2_O. The residual phenol, nucleic acids, and proteins were removed by dialysis against ddH_2_O and treatment by Dnase I, RNase A, or proteinase K, respectively. SDS-PAGE and agarose gel electrophoreses were performed to examine protein and/or nucleic acid contamination, respectively. The purified LPS was re-suspended in ddH_2_O, and its concentration was determined by using the anthrone-sulfuric acid method [39].

The O-specific polysaccharide from LPS was prepared by hydrolyzing LPS with 1% acetic acid at 100 °C for 2 h. The precipitated lipid A was removed by low-speed centrifugation at 524 × g for 30 min [40,41]. Water-soluble fractions We showcould double-distilled H_2_O for 2 d in dialysis tubes (MWCO 3,500) to remove acetic acid.

### Inhibition of the hydrolytic activity of lysozyme

The inhibition of lysozyme activity by LPS or the O-specific polysaccharide was determined as described by Callewaert [14] using *M. lysodeikticus* ATCC 4698 (Sigma) and lysozyme from chicken egg white (Sigma).

### Gel filtration chromatography

Gel filtration chromatography was carried out using an Enrich SEC 650 (10/300) column (Bio Rad) [18,22]. For analysis, 500 μL of lysozyme (500 μg/mL) and 500 μL of LPS (500 μg/mL) were mixed and then incubated for 30 min at 37 °C at room temperature, and applied to the column at a flow rate of 1.0 mL/min. The elution profile was analyzed by monitoring *A*215, and by SDS-PAGE and western blot analyses of the fractions using an affinity-purified anti-lysozyme antibody (1:500 dilution, ab391, Abcam).

### Statistical analysis

Statistical analyses were performed using Prism 5.0 (GraphPad). One-way ANOVA was performed to analyze the *in vitro* killing assay data and for membrane integrity determination. Differences were deemed to be statistically significant at *P* < 0.05.

## Supplementary Material

1433979.zip
