# Supplementary material for: O-specific polysaccharide confers lysozyme resistance to extraintestinal pathogenic Escherichia coli
Source: Virulence. 2018 Mar 19;9(1):666–80. doi: 10.1080/21505594.2018.1433979 (PMC5955474; doi:10.1080/21505594.2018.1433979)
Supplement: 1433979.zip [file kvir-09-01-1433979-s001.zip › 1433979/2017VIRULENCE0239R2-s02.docx]

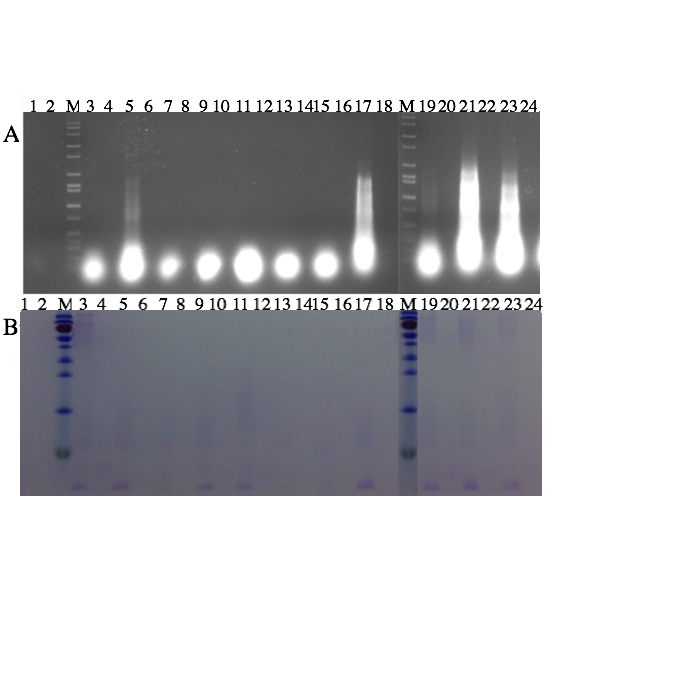


**Figure S1** The purity of LPS isolated from wild type，mutants and complemented strains before (lanes 1, 3, 5, 7, 9, 11, 13, 15, 17, 19, 21, 23) and after (lanes 2, 4, 6, 8, 10, 12, 14, 16, 18, 20, 22, 24) purification A: [agarose gel electrophoresis](http://www.baidu.com/link?url=xwizsIrjXU3cIoi-SdyAUI_6AIxl0Imm1T74pIf_7mT8NgxZHddbUv8PNZuf8s_v3HGRvw8rp1o-y67GAsv1tKVI1SBXydWGZU1MU4olRPBnnbC9qeX0ATkycJZAO8EeUVL8KURZAe316yr64wECAK&wd=&eqid=ffe7f04b000224ea00000005599ebde5); B: SDS-PAGE with coomassie blue staining. 1-2: MG1655, 3-4: NMEC38, 5-6: N380, 7-8: N381, 9-10: N383, 11-12: N384, 13-14: N385, 15-16: N380C, 17-18: N381C, 19-20: N383C, 21-22: N384C, 23-24: N385C, M: DNA Marker 5000 (A) or protein prestained Marker (B).
