# Supplementary material for: O-specific polysaccharide confers lysozyme resistance to extraintestinal pathogenic Escherichia coli
Source: Virulence. 2018 Mar 19;9(1):666–80. doi: 10.1080/21505594.2018.1433979 (PMC5955474; doi:10.1080/21505594.2018.1433979)
Supplement: 1433979.zip [file kvir-09-01-1433979-s001.zip › 1433979/2017VIRULENCE0239R2-s03.docx]

**Identification of the transposon insertion sites and sequence analyses**

Construction of a transposon mutant library of NMEC38

A pool of pUTmini-Tn5*km2* plasmids (Amp^R^, Km^R^) was kindly provided by David Holden (Imperial College, London, UK). Plasmid pUTmini-Tn5*km2* was transformed into donor strain *E. coli* S17-1 λ*pir*. A spontaneous nalidixic acid-resistant mutant of NMEC38 was generated by growing NMEC38 in the presence of the antibiotic and plating 10^9^ cfu to solid media containing nalidixic acid and confirmed to retain full resistance to lysozyme. To construct transposon mutants of NMEC, 400 μl donor and 400 μl recipient cells were mixed and centrifuged. After discarding the supernatant the cells were resuspended in 10 μl of a 10 ^mM^ MgSO4 solution and plated onto LB agar plates and incubated at 37°C for 8 h. Colonies were collected and resuspended in PBS and plated onto LB agar containing kanamycin and nalidixic acid. Following selective overnight growth, single colonies were resuspended in 300 μl LB supplemented with nalidixic acid and kanamycin. After an overnight incubation, 53 μl glycerol (15%) was added and a total of 15000 mutants were stored at -70 ºC in microtiter plates.

**Identification of NMEC38 mutants with decreased resistance to lysozyme**

All mutants were inoculated in 96-well, U-bottom plates containing 1 mL of sterile LB medium and grown at 37 °C to late exponential phase (OD600 = 2.0). After centrifugation at 4,000 × g for 10 min, the bacterial pellets were washed twice with PBS and re-suspended in 1 mL of PBS. Next, 50 μL of the suspensions were transferred to new 96-well plates, and 50 μL of lysozyme solution (12.5 mg/mL, Sigma-Aldrich) was added. The mixtures were incubated at 37 °C for 12 h. The lysed mutant strains were selected for gene identification.

**Identification and characterization of the interrupted genes.**

Transposon insertion sites were amplified by arbitrary PCR. For the first round, the arbitrary primers Arbi5 (GGCCACGCGTCGACTAGTAC(N)10TACNG) were used in combination with transposon-specific primer P9 (CGCAGGGCTTTATTGATTC). Subsequently, 1 µl of each PCR product was used in a second round of nested PCR with primers Arbi2 and P6 (CCTAGGCGGCCAGATCTGAT). Arbi2 (GGCCACGCGTCGACTAGTAC) is homologous to the 5’-sequences of the above mentioned arbitrary primers and P6 is a transposon I termini-specific primer. Genomic DNA from the wild-type strain served as negative control in all reactions. The second round PCR products were purified and sequenced. DNA sequencing was performed in the DNA Facility at Iowa State University. Data analysis was performed using public DNA and protein databases (<http://www.ncbi.nlm.nih.gov/>BLAST) and employing the BLASTX and BLASTN algorithms (1). All DNA sequences were compared with sequences in the TIGR comprehensive microbial resource.

The PCR reaction:

| 5.0 µl | 10×PCR buffer |
| --- | --- |
| 0.2 mM each | dNTPs (100 mM) |
| 1.5 mM | MgCl_2_ (50 mM) |
| 10 pM each | primers |
| 0.05 U/µl | Golden AmpTag |
| 5 µg genomic DNA/ 1µl PCR production | templater |
| up to 50 µl | distilled water |

The first round of PCR was performed:

| 95 ºC | 8 min | 1× |
| --- | --- | --- |
| 95 ºC | 45 s |  |
| 30 ºC | 30 s | 6× |
| 72 ºC | 1.5 min (5 sec/cycle more) |  |
| 95 ºC | 45 s |  |
| 45 ºC | 30 s | 30× |
| 72 ºC | 2 min (5 sec/cycle more) |  |
| 72 ºC | 10 min | 1× |

The second round of PCR was performed:

| 95 ºC | 8 min | 1× |
| --- | --- | --- |
| 95 ºC | 45s |  |
| 55 ºC | 45s | 30× |
| 72 ºC | 1.5min (5s/cycle more) |  |
| 72 ºC | 10min | 1× |
